# Serum levels of lipoprotein-associated phospholipase A2 are associated with coronary atherosclerotic plaque progression in diabetic and non-diabetic patients

**DOI:** 10.1186/s12872-024-03931-x

**Published:** 2024-05-14

**Authors:** Shudong Zhang, Jiangang Wang, Shuai Chen, Ying Zhang, Ruming He, Xiaoqun Wang, Fenghua Ding, Wenbo Hu, Yang Dai, Lin Lu, Ruiyan Zhang, Jingwei Ni, Qiujing Chen

**Affiliations:** 1 Department of Cardiovascular Medicine, Wuxi branch of Ruijin Hospital, Shanghai Jiao Tong University School of Medicine, Wuxi, China; 2 Health Management Medicine Center, the Third Xiangya Hospital, Central South University, Changsha, China; 3 Department of Cardiovascular Medicine, Ruijin Hospital, Shanghai Jiao Tong University School of Medicine, 197 Ruijin Road II, Shanghai, 200025 China; 4 Institute of Cardiovascular Diseases, Shanghai Jiao Tong University School of Medicine, Shanghai, China; 5 Eachy biopharma, Zhangjiagang, Jiangsu Province China

**Keywords:** Lp-PLA2, Type 2 diabetes mellitus, Plaque progression

## Abstract

**Background:**

Lp-PLA2 is linked to cardiovascular diseases and poor outcomes, especially in diabetes, as it functions as a pro-inflammatory and oxidative mediator.

**Objectives:**

This research aimed to explore if there is a connection between the serum levels of Lp-PLA2 and the progression of coronary plaques (PP) in individuals with type 2 diabetes mellitus (T2DM) and those without the condition.

**Materials and methods:**

Serum Lp-PLA2 levels were measured in 137 T2DM patients with PP and 137 T2DM patients with no PP, and in 205 non-diabetic patients with PP and 205 non-diabetic patients with no PP. These individuals met the criteria for eligibility and underwent quantitative coronary angiography at the outset and again after about one year of follow-up. The attributes and parameters of the participants at the outset were recorded.

**Results:**

Increased serum levels of Lp-PLA2 were closely associated with coronary artery PP, and also significantly correlated with change of MLD, change of diameter stenosis and change of cumulative coronary obstruction in both diabetic and non-diabetic groups, with higher correlation coefficients in diabetic patients as compared with non-diabetic patients. Moreover, multivariate logistic regression analysis showed that serum Lp-PLA2 level was an independent determinant of PP in both groups, with OR values more significant in diabetic patients than in non-diabetic patients.

**Conclusions:**

Levels of serum Lp-PLA2 show a significant association with the progression of coronary atherosclerotic plaque in patients with T2DM and those without, especially among individuals with diabetes.

## Introduction

Overwhelming evidence has shown that atherosclerosis is a lipid-driven inflammatory disease [1, 2]. Pro-inflammatory mediators dictate the atherogenic process and final clinical outcome [1, 2]. Inflammation contributes to all stages of the atherosclerotic plaque life cycle [3]. Importantly, non-resolving inflammation induces the development of unstable atherosclerotic plaques by promoting sustained plaque pathology and the formation of large necrotic cores and thin fibrous caps, ultimately leading to plaque rupture [4–7]. Hyperglycemia is independently associated with accelerated formation of atherosclerotic lesions in the absence of hyperlipidemia [8].

Lipoprotein-associated phospholipase A2 (Lp-PLA2) is predominantly linked to low-density lipoprotein (LDL) cholesterol. It is secreted by macrophages and has the capability to cleave oxidized fatty acids from oxidized phospholipids in LDL and lipoprotein (Lp) (a). This process produces soluble pro-inflammatory and pro-apoptotic lipid mediators, namely lyso-phosphatidylcholine and oxidized non-esterified fatty acids [9, 10]. This enzyme has been detected in atherosclerotic plaques in humans and confirmed as a potential contributor to atherosclerotic cardiovascular diseases [11]. Elevated Lp-PLA2 activity is linked to an elevated risk of cardiac fatality, heart attack, acute coronary events, and ischemic stroke [9, 12]. Inhibition of Lp-PLA2 attenuates atherogenesis in animal studies [13]. Lp-PLA2 inhibitor reduces oxidative stress and NLRP3 inflammasome activation in macrophages [14, 15].

In the present study, we evaluated serum Lp-PLA2 levels in diabetic and non-diabetic patients with or with no coronary atherosclerotic plaque progression (PP). The relationship between Lp-PLA2 levels and angiographically documented PP was determined in both diabetic and non-diabetic patients using logistic regression analysis.

## Methods

The investigation adhered to the principles delineated in the Declaration of Helsinki, and explicit written consent was acquired from each participant.

### Patients

**Fig. 1:**
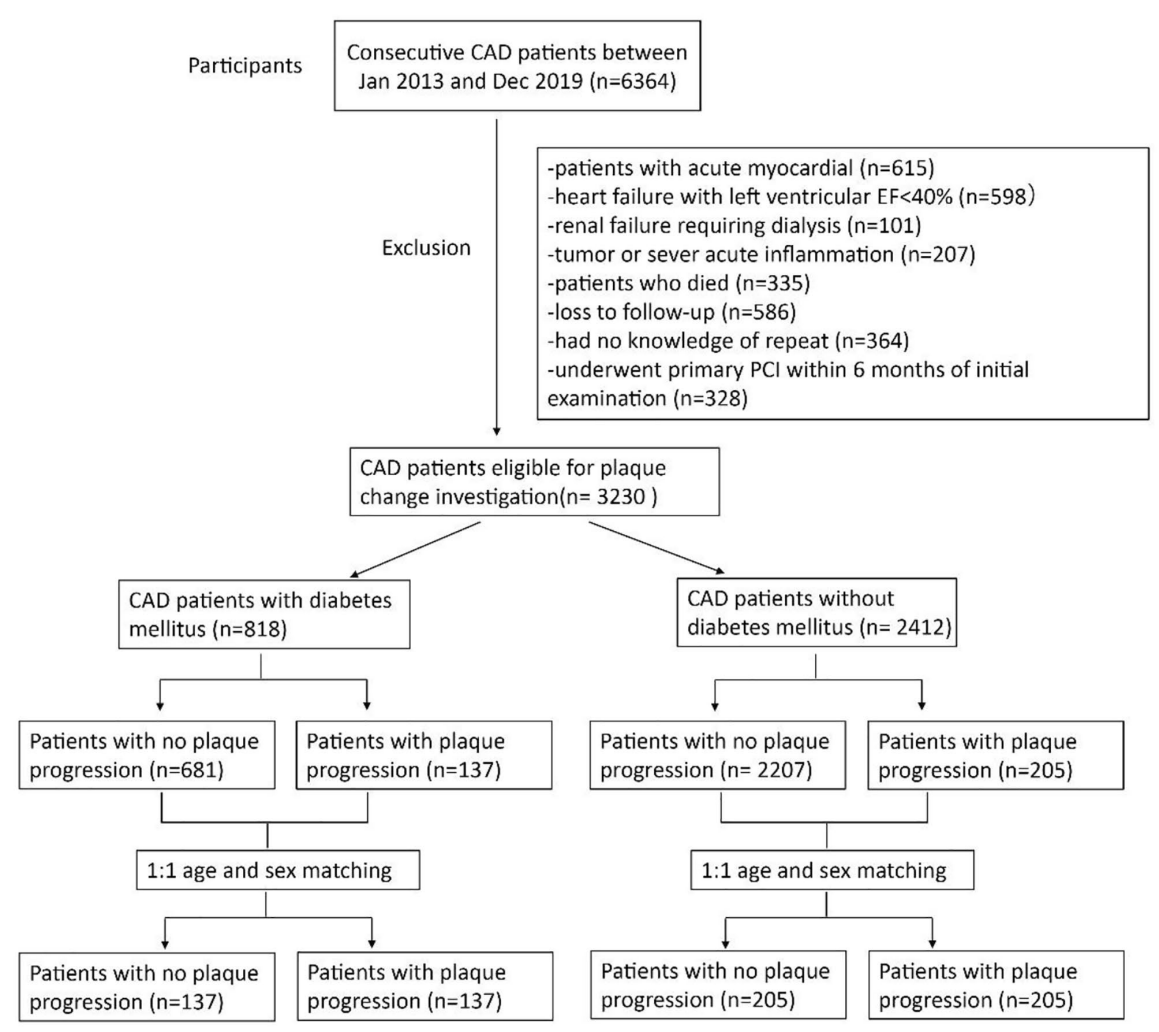
Flow chart of recruitment procedure. CAD: coronary artery disease; EF: ejection fraction; PCI: percutaneous coronary intervention

The individuals enrolled in the study were individuals diagnosed with coronary artery disease (CAD). They were all recruited from Shanghai Ruijin Hospital. From January 2013 to December 2019, a sum of 6364 consecutive patients, who were diagnosed with CAD, received percutaneous coronary intervention (PCI) involving drug-eluting stents (refer to Fig. 1). In this study, patients who underwent primary PCI within six months after the initial angiographic examination were excluded (*n* = 328). Patients who died during the follow-up period (*n* = 335), those lost to follow-up (*n* = 586), and patients without information on repeat angiography (*n* = 364) were all excluded from this study. Additionally, acute myocardial infarction patients (*n* = 615), patients with severe heart failure and a left ventricular ejection fraction < 40% (*n* = 598), renal failure patients requiring dialysis (*n* = 101), and patients with severe acute inflammation or tumors (*n* = 207) were also excluded from this study as well. The remaining potential study population consisted of 3230 CAD patients, with 818 having type 2 diabetes mellitus (T2DM) and 2412 without T2DM. (These patients with diabetes are all type 2 diabetics.) The diagnostic criteria for T2DM are based on the 2021 American Diabetes Association (ADA) diabetes diagnostic standards [16]. Patients with a baseline coronary condition featuring a stenosis (lumen diameter stenosis > 20%) in non-PCI interventional vessels underwent follow-up angiography around one year later.

### Biochemical Assessment

After a night of fasting, blood samples were collected by professional nurses from the patients. The laboratory personnel then separated the serum, which was stored in a -80 °C freezer until needed for testing. The detection of Lp-PLA2 levels utilized a kit provided by Eachy Biopharma (China, Jiangsu) with Registration Certificate for Medical Device number 20,212,400,813. This kit employed a time-resolved fluorescence lateral flow immunoassay (TRFIA) method, incorporating the principles of immunochromatography and a double antibody sandwich. The measurement of patients’ serum glucose, creatinine, blood urea nitrogen (BUN), uric acid, C-reactive protein (CRP) levels, as well as the testing of all lipid parameters, were carried out using the AU5800 automatic biochemistry analyzer from Beckman Coulter (USA). Serum glucose, BUN, uric acid, triglyceride, total cholesterol and CRP levels were measured using Beckman Coulter (USA) reagents with the serial numbers OSR6221, OSR6234, OSR6298, OSR61118, OSR6216 and OSR6199, respectively. Serum LDL-cholesterol and HDL-cholesterol levels were assessed using reagents from Minaris Medical Company (Japan, Tokyo) with catalogue numbers 064357 and 064356, respectively. Serum and urinary creatinine levels were determined utilizing reagents from Kehua Bio-Engineering company (China, Shanghai) with catalogue number 717D. The eGFR was calculated utilizing the Chronic Kidney Disease Epidemiology Collaboration equation [17]. Urinary microalbumin to creatinine ratio (UACR) levels were assessed with the HITACHI 3500 automatic biochemistry analyzer from Japan. Urinary microalbumin levels were measured using reagents from Byron Diagnostics company (China, Shanghai) with catalogue number BYA14120.

### Evaluation of Plaque Progression

Coronary angiography and interventional treatment were performed utilizing either the radial artery approach or the conventional Judkins technique [18]. Nitroglycerin (100 µg) was consistently administered into the coronary artery to prevent arterial spasms. Throughout the initial and subsequent investigations, quantitative coronary artery analysis (QCA) (Centricity Cardiology CA 1000. v1.0, USA) utilized coronary angiography recordings taken from the same projection. Two highly skilled interventional specialists in cardiology, unaware of the patients’ clinical data, analyzed all images.

We selected images from baseline angiograms and follow-up angiograms, collected at the end-diastolic phase of the cardiac cycle and captured from identical angles. These image frames effectively highlight the minimal anterior constriction, overlapping branches, and the most pronounced stenosis. Images were obtained based on the anatomical positions of the distal and proximal branches of the artery, and corresponding arterial segments were delineated. During the follow-up examination, we focused on analyzing segments that exhibited new lesions in arteries without prior PCI, with stenosis levels of ≥ 20% at baseline. Additionally, we examined all plaques with reference diameters equal to or exceeding 1.5 mm. During diastole, the minimum lumen diameter (MLD) was measured from various angles, calibrated using the outer diameter of the catheter filled with contrast agent. Subsequently, we documented the image of the single most severe lesion. We determined the average diameter of the angiographically normal vascular segment, which is 5 millimeters in length and situated between the proximal and distal ends of the affected vessel but excluding any major lateral branches, as the reference diameter. The characteristic of progression in atherosclerotic plaques is the presence of ≥ 1 lesions, with the MLD decreasing to ≥ 0.4 mm from baseline to follow-up angiography (approximately twice the standard deviation of repeated lesion measurements) [19]. The identification features of a newly occurring coronary artery lesion are as follows: no evident stenosis was observed during the previous angiography, or the stenosis diameter of the affected vessel is less than 20%. However, for the follow-up angiography, a reduction in MLD equal to or greater than 0.4 mm was considered significant [20]. The change of coronary artery stenosis score (CCSS) was calculated based on the average MLD across all measured vascular segments for each patient. By summing the percentages of diameter stenosis in coronary arteries, we represent the cumulative occlusion of coronary arteries in standard index units [21]. The changes in QCA measurements were defined as the difference between baseline QCA values and measurements taken during follow-up.

Among the 818 eligible T2DM patients for the study, 137 individuals exhibited coronary atherosclerotic plaque progression (PP). The remaining 681 patients showed no signs of PP. From this group of T2DM patients without PP, 137 consecutive individuals were randomly selected as the control group (no PP). Likewise, among 2412 non-diabetic patients, 205 patients had PP, while 205 consecutive patients of 2207 with no PP were denoted as non-diabetic with no PP group (Fig. 1).

### Statistical analyses

We conducted statistical analyses using SPSS for Windows version 23.0 (SPSS Inc., USA). A two-tailed P value less than 0.05 was deemed statistically significant. The assessment of normal distribution was conducted using the Kolmogorov-Smirnov test. In instances of normal distribution, continuous variables were presented as mean ± standard deviation (SD); otherwise, they were presented as the median (25th – 75th percentile). Categorical variables were exhibited as frequencies (percentages), and group comparisons were made using the chi-square test. In cases requiring statistical calculation for normal distribution, logarithmic transformation was applied to continuous variables with non-normal distribution. Intergroup comparisons were performed using the unpaired t-test, ANOVA, or the nonparametric Mann-Whitney U test when appropriate. Spearman’s correlations were employed to assess relationships between continuous variables (e.g., Lp-PLA2 level) and changes in MLD, stenosis diameter, and CCSS. Multivariable logistic regression models were employed to estimate independent risk factors for plaque progression.

## Results

### Characteristics of the patients

The baseline characteristics and parameters of the participants are detailed in Table 1. In both diabetic and non-diabetic groups, as expected, significant difference revealed between patients with PP and those with no PP regarding parameters indicating coronary atherosclerotic plaque alterations, such as changes in MLD, changes in stenosis diameter, and changes of CCSS. There was no statistical difference between patients with PP and those with no PP in terms of gender, age, body mass index, serum levels of total cholesterol, LDL-cholesterol, HDL-cholesterol, triglyceride, apolipoprotein A, apolipoprotein B, lipoprotein(a), HbA1c, BUN, creatinine, uric acid, UACR and the ratio of medications. However, compared with patients with no PP, both diabetic and non-diabetic patients with PP had significantly higher rates of cigarette smoking and hypertension ratio, and increased hsCRP levels.

**Table 1:** Baseline parameters of the patients

	Diabetes mellitus			Non-diabetes mellitus		
	No PP (*n* = 137)	PP (*n* = 137)	*P* value	No PP (*n* = 205)	PP (*n* = 205)	*P* value
Male, n (%)	98(71.5)	98(71.5)	1.000	152(74.1)	152(74.1)	1.000
Age, years	66.23 ± 9.40	66.51 ± 9.83	0.807	64.92 ± 9.05	65.11 ± 9.24	0.833
Body mass index, Kg/m^2^	24.74 ± 2.88	25.27 ± 3.39	0.170	25.24 ± 3.27	24.95 ± 3.38	0.377
Cigarette smoking, n (%)	32(23.4)	55(40.1)	0.003	49(23.9)	73(35.6)	0.010
Hypertension, n (%)	85(62.0)	108(78.8)	0.002	138(67.3)	161(78.5)	0.011
Hyperlipidemia, (%)	23(16.8)	28(20.4)	0.438	31(15.1)	29(14.1)	0.780
Systolic blood pressure, mm Hg	137.67 ± 18.96	139.28 ± 18.98	0.482	137.25 ± 19.61	137.59 ± 19.06	0.860
Diastolic blood pressure, mm Hg	75.66 ± 13.09	75.70 ± 11.04	0.980	75.63 ± 11.29	75.41 ± 10.15	0.836
Fasting blood glucose, mmol/L	7.09 ± 1.81	7.05 ± 2.32	0.867	5.40 ± 0.75	5.38 ± 0.70	0.838
HbA1c, %	7.38 ± 1.26	7.42 ± 1.42	0.818	5.84 ± 0.46	5.79 ± 0.49	0.324
Serum creatinine, µmol/L	78.60 ± 21.49	81.75 ± 24.31	0.256	80.00 ± 17.89	81.83 ± 15.91	0.276
Serum BUN, mmol/L	6.71 ± 2.49	6.77 ± 3.75	0.882	6.49 ± 2.45	6.70 ± 2.87	0.430
Serum uric acid, µmol/L	332.75 ± 81.50	345.21 ± 92.38	0.237	346.18 ± 89.17	347.76 ± 93.53	0.861
UACR, mg/g	2.50(2.40–3.60)	2.50(2.50–4.79)	0.370	2.50(2.50–2.50)	2.50(2.50–2.85)	0.146
eGFR, mL/min/1.73m^2^	82.81 ± 17.23	80.40 ± 18.42	0.264	83.60 ± 19.89	81.21 ± 22.04	0.251
Triglyceride, mmol/L	1.65 ± 0.86	1.61 ± 1.00	0.702	1.61 ± 1.19	1.64 ± 1.08	0.779
Total cholesterol, mmol/L	3.83 ± 1.18	3.91 ± 1.16	0.578	3.89 ± 1.01	3.94 ± 1.08	0.622
HDL cholesterol, mmol/L	1.07 ± 0.28	1.06 ± 0.26	0.608	1.13 ± 0.28	1.10 ± 0.25	0.242
LDL cholesterol, mmol/L	2.16 ± 0.88	2.36 ± 0.94	0.073	2.30 ± 0.92	2.33 ± 0.88	0.688
Apolipoprotein A, g/L	1.23 ± 0.22	1.20 ± 0.23	0.308	1.25 ± 0.23	1.23 ± 0.21	0.335
Apolipoprotein B, g/L	0.74 ± 0.21	0.78 ± 0.24	0.214	0.74 ± 0.22	0.77 ± 0.29	0.287
Lipoprotein (a), g/L	0.24 ± 0.27	0.25 ± 0.26	0.713	0.27 ± 0.24	0.25 ± 0.24	0.320
hsCRP, mg/L	0.73(0.34–2.14)	1.04(0.48–3.90)	0.007	0.79(0.32–1.60)	0.97(0.44–3.73)	0.002
Medication, n (%)						
ACE inhibitors/ARBs	86(62.8)	94(68.6)	0.309	129(62.9)	134(65.4)	0.607
β-blockers	72(52.6)	80(58.4)	0.331	130(63.4)	124(60.5)	0.542
Statins	128(93.4)	125(91.2)	0.496	194(94.6)	192(93.7)	0.674
Metformin, (%)	47(34.3)	45(32.8)	0.798	/	/	/
Insulin, (%)	32(23.4)	29(21.2)	0.663	/	/	/
LP-PLA2, (ng/mL)	130.11 ± 71.34*	203.17 ± 147.63*^##^	< 0.001	125.22 ± 91.08	169.68 ± 118.67	< 0.001
Follow-up						
Duration, months	12.25 ± 0.91	12.29 ± 0.90	0.688	12.11 ± 0.88	12.18 ± 0.91	0.408
Changes in MLD, (mm)	-0.01 ± 0.14**	0.69 ± 0.25**^##^	< 0.001	-0.04 ± 0.11	0.57 ± 0.18	< 0.001
Changes in stenosis diameter (%)	-0.09 ± 3.94**	-23.22 ± 9.70**^##^	< 0.001	1.16 ± 2.28	-18.19 ± 3.58	< 0.001
Changes in CCSS	-0.03 ± 0.09**^#^	-0.34 ± 0.15**^##^	< 0.001	-0.01 ± 0.06	-0.22 ± 0.04	< 0.001

Values are given as mean ± standard deviation (SD), median (25th–75th percentile) or number (percentage)

Abbreviation: ACE, angiotensin converting enzyme; ARB, angiotensin receptor blocker; BUN, blood urea nitrogen; UACR, urine albumin to creatinine ratio; CRP, C-reactive protein; eGFR, estimated glomerular filtration rate; HbA1c, glycosylated hemoglobin; HDL, high-density lipoprotein; LDL, low-density lipoprotein; MLD, minimal lumen diameter; CCSS, cumulative coronary stenosis score

*P < 0.05, vs. PP in non-diabetes mellitus group

**P < 0.001, vs. PP in non-diabetes mellitus group

^#^*P* < 0.05, vs. No PP in non-diabetes mellitus group

^##^*P* < 0.001, vs. No PP in non-diabetes mellitus group

### Association of serum Lp-PLA2 levels with plaque progression in diabetic and non-diabetic patients

To ascertain the potential relation of Lp-PLA2 with PP, we evaluated serum levels of Lp-PLA2 in all the patients. The results showed that serum levels of Lp-PLA2 were significantly increased in patients with PP than in those with no PP in both diabetic and non-diabetic groups (diabetic group, 203.17 ± 147.63 vs. 130.11 ± 71.34, *P* < 0.001; non-diabetic group, 169.68 ± 118.67 vs. 125.22 ± 91.08, < 0.001, respectively) (Table 1). Furthermore, serum levels of Lp-PLA2 were correlated with changes in MLD, changes in stenosis diameter, and changes in CCSS (all *P* < 0.001), with higher correlation coefficients observed in the diabetic group compared to the non-diabetic group (Table 2). We further categorized the diabetic and non-diabetic patients respectively into tertile subgroups according to Lp-PLA2 levels (Table 3). Significant difference in PP ratio, alterations in MLD, stenosis diameter, and CCSS was noted among the tertile subgroups of Lp-PLA2 concentrations (all *P* < 0.01) (Table 3). The diagnostic sensitivity and specificity of serum Lp-PLA2 for PP in diabetic patients were 62.04% and 67.15%, respectively, when the cutoff value was 133.55 ng/mL. For non-diabetic patients, the diagnostic sensitivity and specificity of serum Lp-PLA2 for PP were 42.90% and 79.50%, respectively, when the cutoff value was 169.30 ng/mL.

**Table 2:** Correlation of serum Lp-PLA2 levels with plaque progression in diabetic and non-diabetic patients with CAD.

	Serum levels of Lp-PLA2, ng/mL				
	Diabetes mellitus			Non-diabetes mellitus	
	r	*P* value		r	*P* value
Changes in MLD	0.406	< 0.001		0.222	< 0.001
Changes in stenosis diameter	-0.404	< 0.001		-0.243	< 0.001
Changes in CCSS	-0.363	< 0.001		-0.230	< 0.001

Change of QCA measurement is defined as baseline QCA measurement minus follow-up measurement

Abbreviation: CCSS, cumulative coronary stenosis score; MLD, minimal lumen diameter; QCA, quantitative coronary analyses

**Table 3:** Plaque progression in different tertiles of Lp-PLA2 levels

Tertiles of Lp-PLA2 levels	T1	T2	T3	*P* value
Diabetes mellitus	(*n* = 91)	(*n* = 92)	(*n* = 91)	
PP, n(%)	36(39.6)	36(39.1)	65(71.4)	< 0.001
Changes in MLD, (mm)	0.23 ± 0.37	0.28 ± 0.38	0.52 ± 0.41	< 0.001
Changes in stenosis diameter, (%)	-8.39 ± 13.09	-9.57 ± 12.78	-17.00 ± 13.85	< 0.001
Changes in CCSS	-0.13 ± 0.19	-0.16 ± 0.19	-0.26 ± 0.21	< 0.001
Non-Diabetes mellitus	(*n* = 136)	(*n* = 138)	(*n* = 136)	
PP, n(%)	47(34.6)	69(50.0)	89(65.4)	< 0.001
Changes in MLD, (mm)	0.16 ± 0.31	0.25 ± 0.32	0.38 ± 0.35	< 0.001
Changes in stenosis diameter, (%)	-5.31 ± 9.23	-8.29 ± 10.10	-11.94 ± 10.02	< 0.001
Changes in CCSS	-0.08 ± 0.12	-0.11 ± 0.11	-0.15 ± 0.12	< 0.001

Values are given as mean ± standard deviation (SD) or number (percentage). Change of QCA measurement was defined as baseline QCA measurement minus follow-up measurement

Abbreviation: QCA, quantitative coronary analyses; MLD, minimal lumen diameter; CCSS, cumulative coronary stenosis score

Tertiles of Lp-PLA2 levels in Diabetes mellitus group as: T1≤113.62ng/mL; 113.62 < T2≤171.41ng/mL; T3 > 171.41ng/mL

Tertiles of Lp-PLA2 levels in Non-diabetes mellitus group as: T1≤88.29ng/mL; 88.29 < T2≤162.51ng/mL; T3 > 162.51ng/mL

### Multivariable logistic regression analysis

Multivariable logistic regression analysis was conducted to identify the factors contributing to PP in both diabetic and non-diabetic individuals, encompassing all variables listed in Table 1. The findings indicated that smoking and hypertension independently influenced PP (Model 1). hsCRP independently influenced PP in non-diabetic group (Model 1). Upon introducing Lp-PLA2 levels and controlling for these variables (Model 2), Lp-PLA2 levels maintained an independent association with PP. Lp-PLA2 levels of diabetic group in Model 2 displayed OR for T2 to be 2.09 (95% CI 1.03 ∼ 4.23, *P* < 0.05), and OR for T3 to be 6.78 (95% CI 3.18 ∼ 14.46, *P* < 0.001). Lp-PLA2 levels of non-diabetic group in Model 2 exhibited OR for T2 to be 1.73 (95% CI 1.04 ∼ 2.87, *P* < 0.05), and OR for T3 to be 3.27 (95% CI 1.97 ∼ 5.43, *P* < 0.001) (Table 4), with these OR value lower than those in diabetic group. In contrast to Model 1, the inclusion of Lp-PLA2 levels led to a significant enhancement in the C statistic by 0.070 within Model 2 for the diabetic cohort [rising from 0.684 (95% CI 0.621 ∼ 0.746) to 0.754 (95% CI 0.697 ∼ 0.811)], and by 0.052 within Model 2 for the non-diabetic group [increasing from 0.652 (95% CI 0.599 ∼ 0.705) to 0.704 (95% CI 0.654 ∼ 0.754)].

**Table 4:** Multivariable regression analysis of independent determinants for plaque progression

		Diabetes mellitus			Non-diabetes mellitus	
	Variables	Adjusted OR (95%CI)	*P Value*		Adjusted OR (95%CI)	*P Value*
Model1	Body mass index	1.04 (0.96–1.13)	0.294		0.96 (0.90–1.02)	0.155
	Cigarette smoking	2.05 (1.18–3.56)	0.011		1.82 (1.16–2.85)	0.009
	Hypertension	1.92 (1.09–3.37)	0.024		1.91 (1.20–3.05)	0.007
	UACR, mg/g	1.00 (0.99-1.00)	0.514		1.00 (0.99–1.01)	0.927
	eGFR, mL/min/1.73m^2^	0.99 (0.98–1.01)	0.194		0.99 (0.99-1.00)	0.262
	HDL cholesterol, mmol/L	0.75 (0.29–1.93)	0.555		0.66 (0.30–1.43)	0.289
	LDL cholesterol, mmol/L	1.30 (0.98–1.74)	0.073		1.04 (0.83–1.30)	0.768
	Log-transferred hsCRP	1.53 (1.00-2.36)	0.052		2.02 (1.41–2.90)	< 0.001
	Statins	0.72 (0.28–1.86)	0.500		0.69 (0.29–1.64)	0.403
Model2	Body mass index	1.04 (0.96–1.13)	0.349		0.96 (0.90–1.02)	0.210
	Cigarette smoking	1.87 (1.04–3.36)	0.035		1.61 (1.01–2.55)	0.044
	Hypertension	2.17 (1.19–3.98)	0.012		1.84 (1.14–2.98)	0.013
	UACR, mg/g	1.00 (0.99-1.00)	0.292		1.00 (0.99–1.01)	0.878
	eGFR, mL/min/1.73m^2^	0.99 (0.97-1.00)	0.141		1.00 (0.99–1.01)	0.335
	HDL cholesterol, mmol/L	0.92 (0.34–2.53)	0.877		0.51 (0.23–1.15)	0.106
	LDL cholesterol, mmol/L	1.16 (0.85–1.57)	0.351		1.00 (0.79–1.27)	0.992
	Log-transferred hsCRP	1.67 (1.06–2.63)	0.027		1.99 (1.37–2.88)	< 0.001
	Statins	0.76 (0.28–2.06)	0.591		0.83(0.34–2.03)	0.682
	Tertiles of Lp-PLA2 level		< 0.001			< 0.001
	T1	1(reference)	/		1(reference)	/
	T2	2.06 (1.01–4.19)	0.046		1.72 (1.03–2.86)	0.037
	T3	7.01 (3.27–15.04)	< 0.001		3.27 (1.97–5.43)	< 0.001

Abbreviation: UACR, urine albumin to creatinine ratio; CRP, C-reactive protein; eGFR, estimated glomerular filtration rate; HDL, high-density lipoprotein; LDL, low-density lipoprotein

Tertiles of Lp-PLA2 levels in all patients: T1≤101.32ng/mL; 101.32 < T2≤166.38ng/mL; T3 > 166.38ng/mL

## Discussion

Lp-PLA2 functions as a pro-inflammatory and oxidative mediator. Previous studies have shown an association between heightened Lp-PLA2 activity and an elevated likelihood of acute coronary syndromes, myocardial infarction, fatal cardiac events, and ischemic stroke [9, 12]. In 2014, the Food and Drug Administration sanctioned the Lp-PLA2 examination for individuals lacking prior coronary disease as a means to assess the likelihood of heart disease, cardiac arrest, and potential additional cardiac issues [22]. In a study involving animals, the suppression of Lp-PLA2 has been shown to mitigate the development of atherosclerosis [13]. The comprehensive measurement index of the mass and activity of Lp-PLA2 were found to be linked to the occurrence of CAD in a cohort comprising 224 African Americans and 336 Caucasians who underwent coronary angiography [23]. The activity of Lp-PLA2 exhibited a significant positive correlation with carotid intima-media thickness (IMT) and plaques among a cohort of 929 Japanese elderly men. However, the Mendelian randomized study failed to substantiate Lp-PLA2 as a causative factor for subclinical atherosclerosis [24]. Meanwhile, there are also some contradictory results. In a study examining loss-of-function variants of Lp-PLA2 in a cohort of 90,000 Chinese adults, reduced Lp-PLA2 activity did not demonstrate significant associations with major risks of either vascular or non-vascular diseases [25]. In 2014, Darapladib, developed by GlaxoSmithKline (GSK) and touted as the leading Lp‐PLA2 inhibitor in clinical trials, failed to meet the primary endpoints in two phase III trials involving patients with atherosclerosis [26, 27]. Currently, there have been no reported studies assessing the relationship between serum Lp-PLA2 levels and the coronary artery PP in patients with established CAD, especially in patients with T2DM. Our research has for the first time uncovered a connection between elevated Lp-PLA2 levels in the bloodstream and coronary artery PP. Additionally, these levels showed a correlation with alterations in MLD, variations in stenosis diameter, and changes in CCSS within both diabetic and non-diabetic cohorts. Notably, the correlation coefficients were higher in the diabetic group compared to the non-diabetic group. Lp-PLA2 exhibits a strong connection with the development and advancement of DM microvascular complications [28]. Furthermore, when contrasted with other complications stemming from diabetes, the link between diabetic nephropathy (DN) and Lp-PLA2 stands out as notably prominent [29]. Recent research findings additionally indicate that Lp-PLA2 operates as an autonomous variable when evaluating early DN, potentially serving as a crucially specific marker for diagnosing this condition in its initial stages [30]. Therefore, following literature references, we specifically incorporate UACR into multivariate logistic regression analysis for calibration. The multivariate logistic regression analysis demonstrated that the serum Lp-PLA2 level independently determined PP in both groups, with OR values being more significant in the diabetic group compared to the non-diabetic group. These results suggest that serum Lp-PLA2 level is a contributor to coronary artery PP, especially among diabetic patients.

The atherosclerotic plaque potentially associated with cardiovascular events has specific characteristics. Pathological examinations in autopsy have demonstrated that such plaque contains a lipid-rich necrotic core, and macrophages and smooth muscle cells of robust inflammation [31, 32]. Enhanced inflammation induces activation and chemotaxis of monocytes/macrophages and formation of foam cells, promotes proliferation and differentiation of smooth muscle cells, facilitates the transport of lipids into plaque and impairs revere cholesterol transport in macrophages/foam cells and transdifferentiated smooth muscle cells. These pathological processes lead to atherosclerotic PP and plaque vulnerability, eventually resulting in cardiovascular events [3–7].

Lp-PLA2 is a serine lipase with a specialized function in cleaving fatty acids from oxidized phospholipids, such as those found in oxidized LDL. Circulating Lp-PLA2 secretes mainly by inflammatory cells and associates with the LDL and HDL lipid fractions. Previous studies have shown that serum level of Lp-PLA2 is an independent marker predicting cardiovascular disease risk [33, 34]. The proatherogenic effects of Lp-PLA2 result from its function in producing two crucial proinflammatory mediators, lysophosphatidylcholine and oxidized nonesterified fatty acids, through cleaving oxidized phospholipids. These two inflammatory mediators play a role in attracting monocytes to the arterial wall, matrix-metalloproteinase production, and induction of necrotic core, jointly leading to atherosclerotic plaque development which eventually manifest as PP in coronary angiography.

Moreover, previous studies have exhibited that Lp-PLA2 levels accompany high levels of small dense LDL particles in diabetic patients [35]. Patients with T2DM or poorly glycemic control and high Lp-PLA2 activity have higher risk of major coronary events as compared with those who do not have diabetes or have well-controlled diabetes. Pharmacological inhibition of Lp-PLA2 is most effective in reducing risk of major coronary events for patients with T2DM and high Lp-PLA2 activity, resulting in a 33% reduction in the risk of major coronary events [36]. These results indicate that diabetes could aggravate the impact of Lp-PLA2 on cardiovascular pathology. In this current investigation, there was a connection observed between serum Lp-PLA2 levels and coronary artery PP. Additionally, there were correlations identified with alterations in MLD, variations in stenosis diameter, and changes in CCSS in both the diabetic and non-diabetic cohorts. Notably, the correlation coefficients were higher in the diabetic group compared to those in the non-diabetic group. Multivariable logistic regression analysis also revealed that Lp-PLA2 level was an independent risk factor for PP in both diabetic and non-diabetic patients, with the OR value being higher in the diabetic group than in the non-diabetic group. Collectively, the above-mentioned information consistently suggests a notion that Lp-PLA2 contributes to coronary atherosclerotic PP, especially in patients with T2DM.

### Limitations

We recognize the limitations inherent in our research. Firstly, this investigation is designed as a cross-sectional study, with the primary aim of investigating the correlation between Lp-PLA2 and the advancement of coronary atherosclerotic plaques, without implying causation. Secondly, it would be beneficial if we could include the activity of Lp-PLA2 in the detection as well. Additionally, further research may be needed to explore the potential mechanisms behind this correlation.

## Conclusion

Serum Lp-PLA2 levels are linked to the advancement of coronary atherosclerotic plaques in individuals with T2DM and those without diabetes, implying that Lp-PLA2 plays a vital role as a mediator or marker in the development of atherosclerotic plaques.

## Data Availability

The datasets examined in this research are accessible upon a reasonable request from the corresponding authors.
